# Repeat hepatectomy for liver metastases from bile duct neuroendocrine tumor: a case report

**DOI:** 10.1186/s40792-020-00967-x

**Published:** 2020-08-08

**Authors:** Mamiko Miyashita, Yoshihiro Ono, Manabu Takamatsu, Yosuke Inoue, Takafumi Sato, Hiromichi Ito, Yu Takahashi, Akio Saiura

**Affiliations:** 1 Department of Gastroenterological Surgery, Cancer Institute Hospital, Japanese Foundation for Cancer Research, 3-8-31 Ariake, Koto-ku, Tokyo, 135-8550 Japan; 2 Department of Pathology, Cancer Institute Hospital, Japanese Foundation for Cancer Research, 3-8-31 Ariake, Koto-ku, Tokyo, 135-8550 Japan; 3 Present Address: Department of Hepatobiliary-Pancreatic Surgery, Juntendo University School of Medicine, 2-1-1 Bunkyoku, Hongo, Tokyo, 113-8421 Japan

**Keywords:** Neuroendocrine tumor (NET), Liver metastasis, Repeat hepatectomy, Bile duct

## Abstract

**Background:**

Primary neuroendocrine tumor (NET) originating from the extrahepatic bile duct is rare, although liver metastasis from gastroenteropancreatic NET is frequently observed. We herein report a case who successfully underwent repeat hepatectomy for liver metastases from bile duct NET grade 2 (G2).

**Case presentation:**

A 75-year-old man presented with jaundice and was suspected of perihilar cholangiocarcinoma by computed tomography (CT) and magnetic resonance imaging (MRI). He underwent extended left hepatectomy, extrahepatic bile duct resection, and hepaticojejunostomy. Pathological findings showed a NET G2 of the biliary tract arising from the common bile duct. Two years and 11 months after surgery, a liver metastasis was detected and hepatectomy was performed. During the surgery, another liver metastasis was detected, and limited liver resection for the two lesions was performed. Pathological findings showed four liver metastases of NET G2. Five years and 4 months after the first surgery (2 years and 5 months after the second hepatectomy), four liver metastases were detected. Thereafter, he received somatostatin analogues for 1 year. Although the size of tumors increased slightly, the number did not change. He underwent limited liver resections and was diagnosed with 7 liver metastases of NET G2. Finally, another hepatectomy (fourth hepatectomy) was performed and long-term survival without recurrence was obtained for as long as 8 years after the first surgery.

**Conclusions:**

Repeat hepatectomy is a good option to obtain long-term survival for liver metastases from bile duct NET G2 in select patients.

## Background

A primary neuroendocrine tumor (NET) of the bile duct is rare. It has been reported that NETs originating from the extrahepatic bile duct account for only 0.2–2% of the primary NET sites in gastroenteropancreatic NET [1, 2].

The most common metastatic site of all NETs is the liver. Liver metastasis is an important prognostic factor in patients with NET. In terms of treatment, debulking surgery and radical resection are recommended [3]. There has been no report that details repeat hepatectomy of NET grade 2 (G2) that originated from the bile duct. We herein report a patient with liver metastases from the bile duct NET G2, who successfully underwent hepatectomy three times and recovered 8 years after the first operation.

## Case presentation

**Fig. 1:**
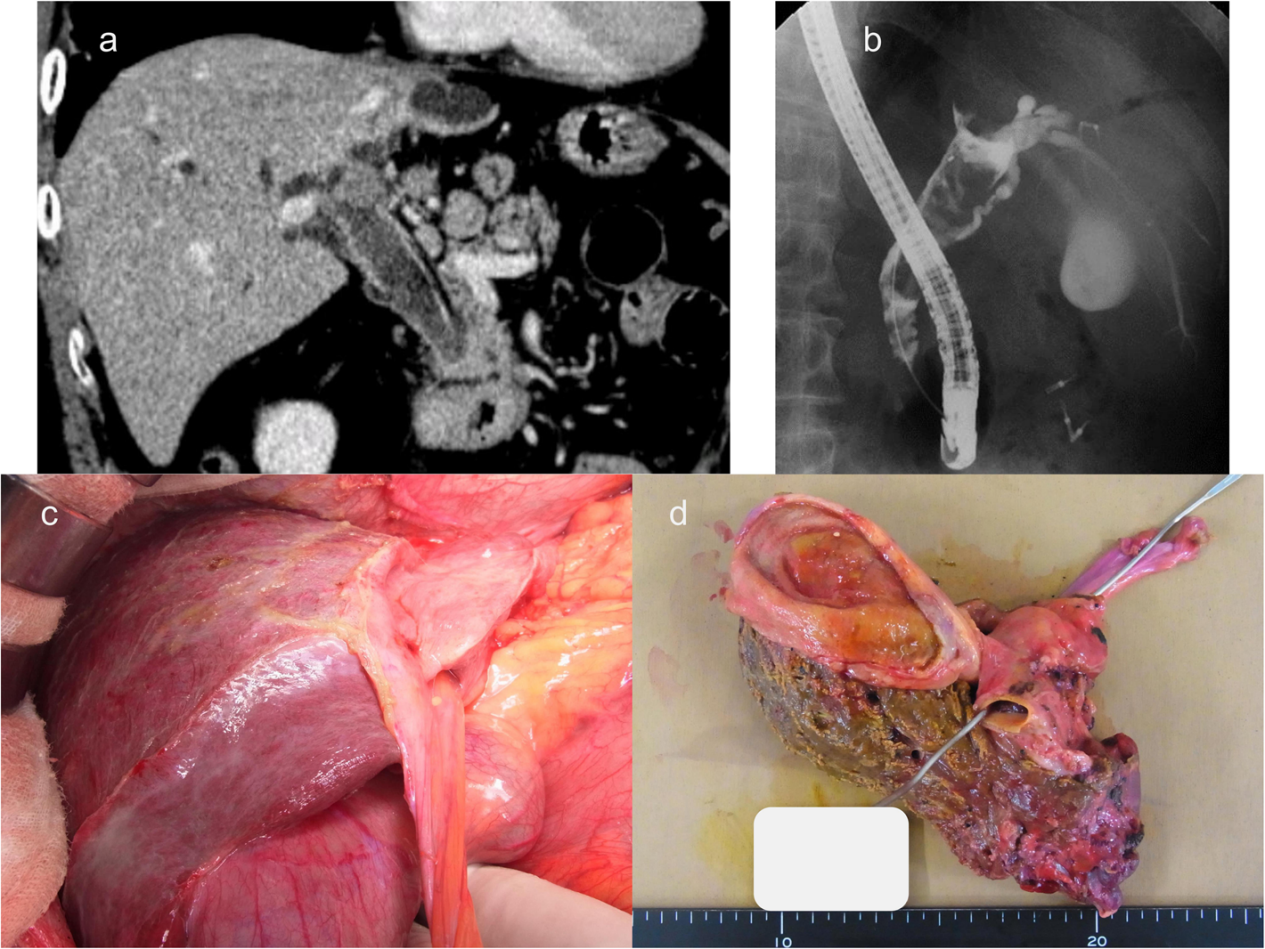
Contrast-enhanced computed tomography (CT) and endoscopic retrograde cholangiopancreatography (ERCP). **a** CT shows dilation of the intrahepatic and extrahepatic bile ducts, wall thickening of the common bile duct, and atrophy of the left liver lobe. **b** ERCP demonstrates a defect in the left intrahepatic and common bile ducts. Furthermore, a mass in the common bile duct is indicated. **c** Intraoperative findings showed atrophy of the left lobe. **d** Left hepatectomy, caudate lobectomy, and extrahepatic bile duct resection were performed

**Fig. 2:**
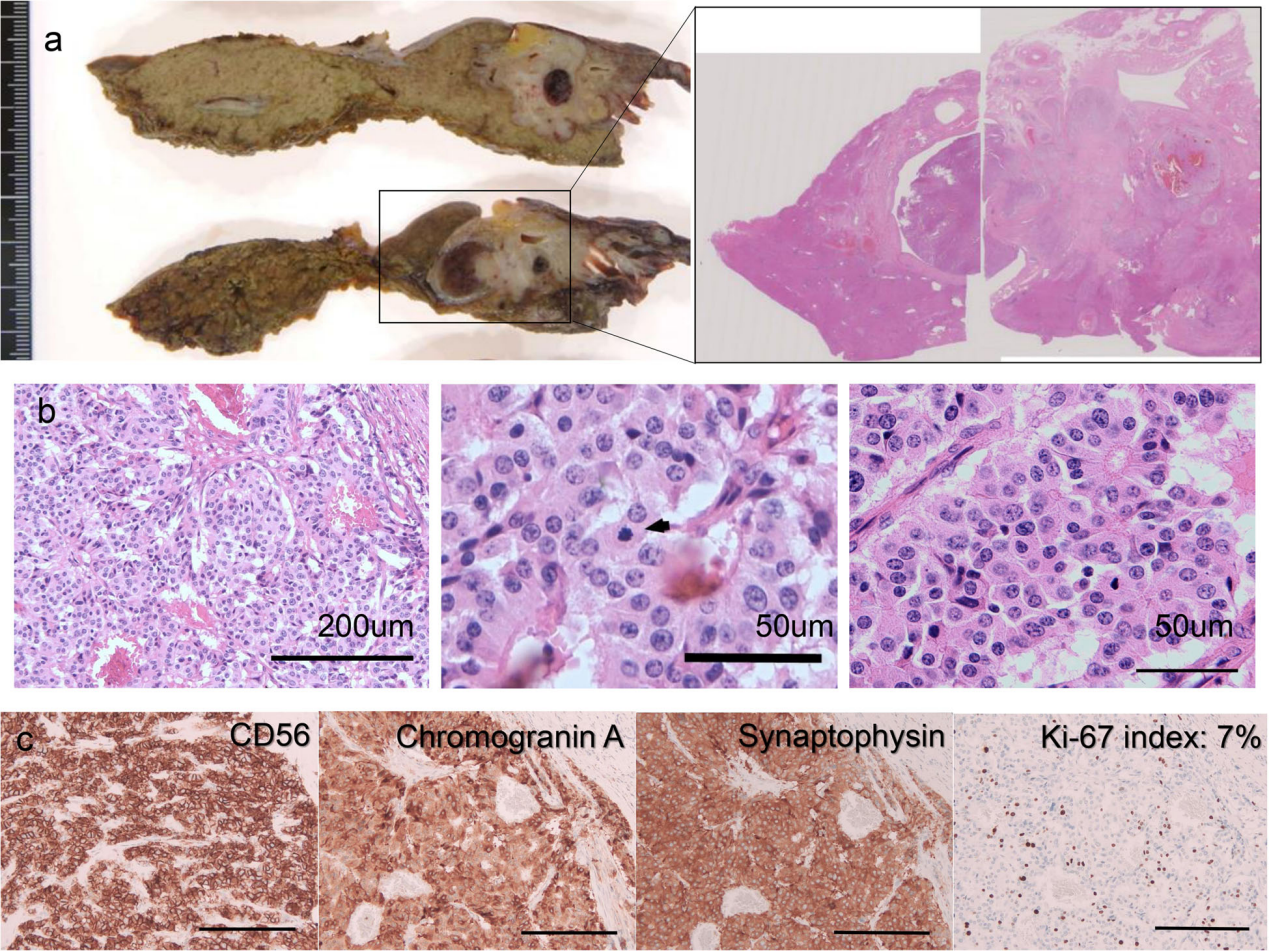
Pathological findings of the initially resected specimen. **a** The tumor arose from the common bile duct and invaded the left hepatic bile duct. **b** Hematoxylin and eosin staining. The tumor nest consists of eosinophilic cells with round nuclei (left). A mitotic figure is indicated by the arrow (middle). Rosette formation is apparent (right). **c** Immunohistochemistry. The tumor cells are positive for all the neuroendocrine markers (CD56, chromogranin A, and synaptophysin). Ki-67 index is 7% (scale bars, 200 μm)

A 75-year-old man presented with upper abdominal pain. The blood biochemical tests showed the following: total bilirubin, 2.3 mg/dL; direct bilirubin, 1.6 mg/dL; gamma-glutamyl transferase (γ-GTP), 194 u/L; alkaline phosphatase (ALP), 1057 u/L; aspartate aminotransferase (AST), 56 u/L; and alanine aminotransferase (ALT), 54 u/L; without abnormalities in amylase. Computed tomography (CT) showed dilation of the intrahepatic and extrahepatic bile ducts, soft tissue density in the common bile duct, wall thickening of the common bile duct, and atrophy of left lobe of the liver (Fig. 1a). Endoscopic retrograde cholangiopancreatography (ERCP) demonstrated defects in the left intrahepatic and common bile ducts (Fig. 1b). Although the cytological analysis obtained from the bile duct did not detect the presence of cancer cells, perihilar cholangiocarcinoma was suspected and the patient underwent left hepatectomy combined with caudate lobectomy and extrahepatic bile duct resection (Fig. 1c, d). Biliary continuity was established by a right hepaticojejunostomy. The operative time was 610 min, and blood loss during surgery was 670 mL. The postoperative course was good, and the patient was discharged 23 days after surgery. The resected specimen showed a whitish and irregularly shaped tumor arising from the common bile duct invading the left hepatic duct (Fig. 2). Histologically, the tumor consisted of well-differentiated neuroendocrine cells forming nest or cord-like pattern. The number of mitoses was 2.4 per 10 high power fields (Fig. 2b). Immunohistochemical staining showed that all the tumor cells were positive for CD56, synaptophysin, and chromogranin A and the Ki-67 index was 7% (Fig. 2c). Collectively, we diagnosed the tumor as NET G2. Surgical margin was negative, and 7 out of 25 lymph nodes were positive for metastasis.

**Fig. 3:**
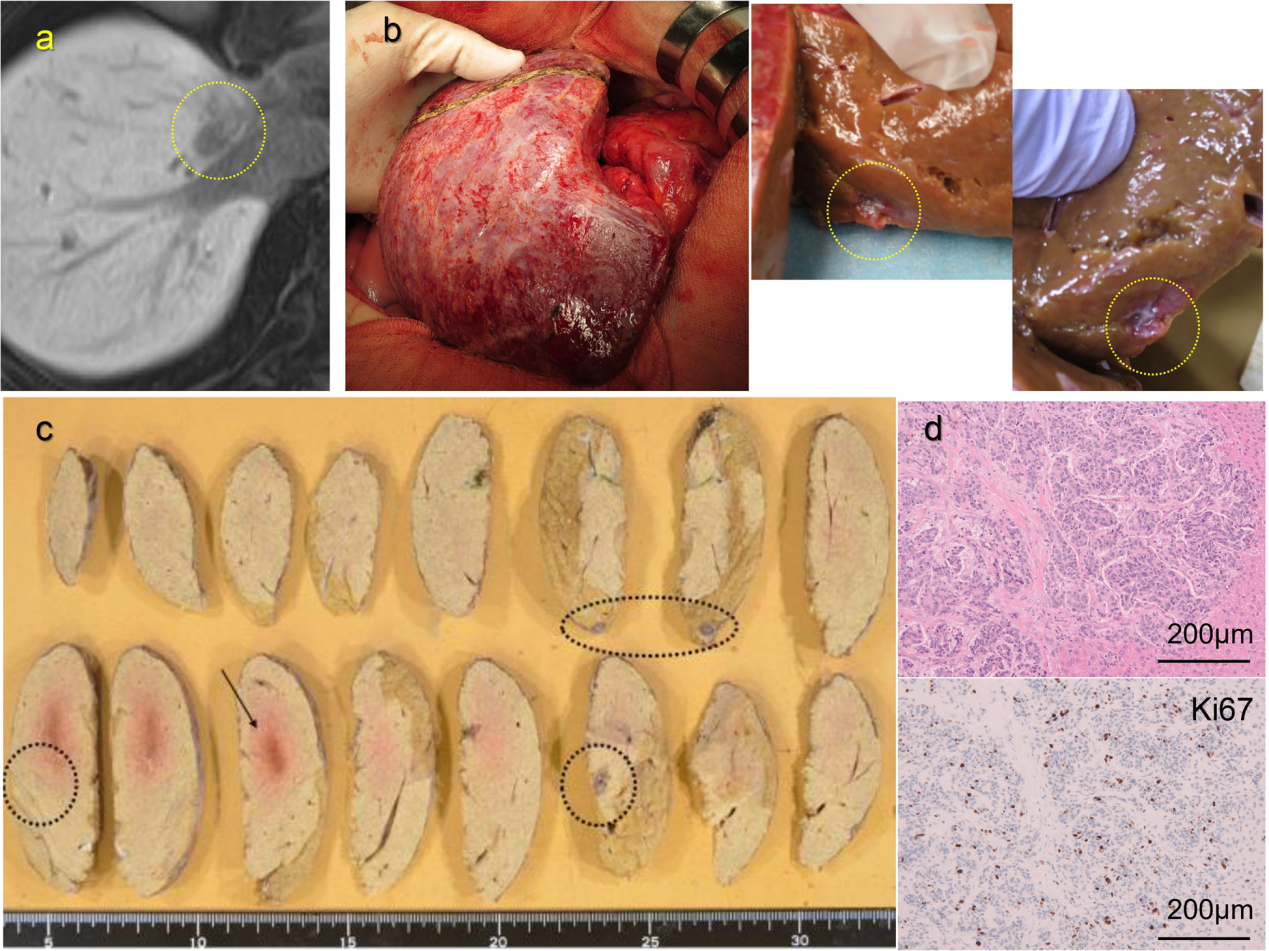
Second operation. **a** CT detected a liver metastasis as a recurrence of NET. **b** Intraoperative findings showed another metastasis. **c** Pathological findings showed four tumors in the resected specimen. The dotted circles represent the metastases newly detected by CE-IOUS or pathological findings. The arrow points the metastasis detected before operation. **d** Histopathological findings were almost the same as the specimen from the last operation

Two years and 9 months after the first operation, a liver metastasis was detected by follow-up CT (Fig. 3a). The patient underwent a limited liver resection of segment 8. Another liver metastasis was newly detected intraoperatively at segment 8 by contrast-enhanced intraoperative ultrasound (CE-IOUS) using perflubutane (Fig. 3b). It was difficult to find this tumor by CT, MRI, or contrast-enhanced ultrasound (CEUS) before surgery. Limited liver resections were performed for these two lesions. Pathological findings showed two other metastases in the resected lesions (Fig. 3c), and a total of four metastases were included in the specimen. All four liver metastases were diagnosed as NET G2 (Fig. 3d).

**Fig. 4:**
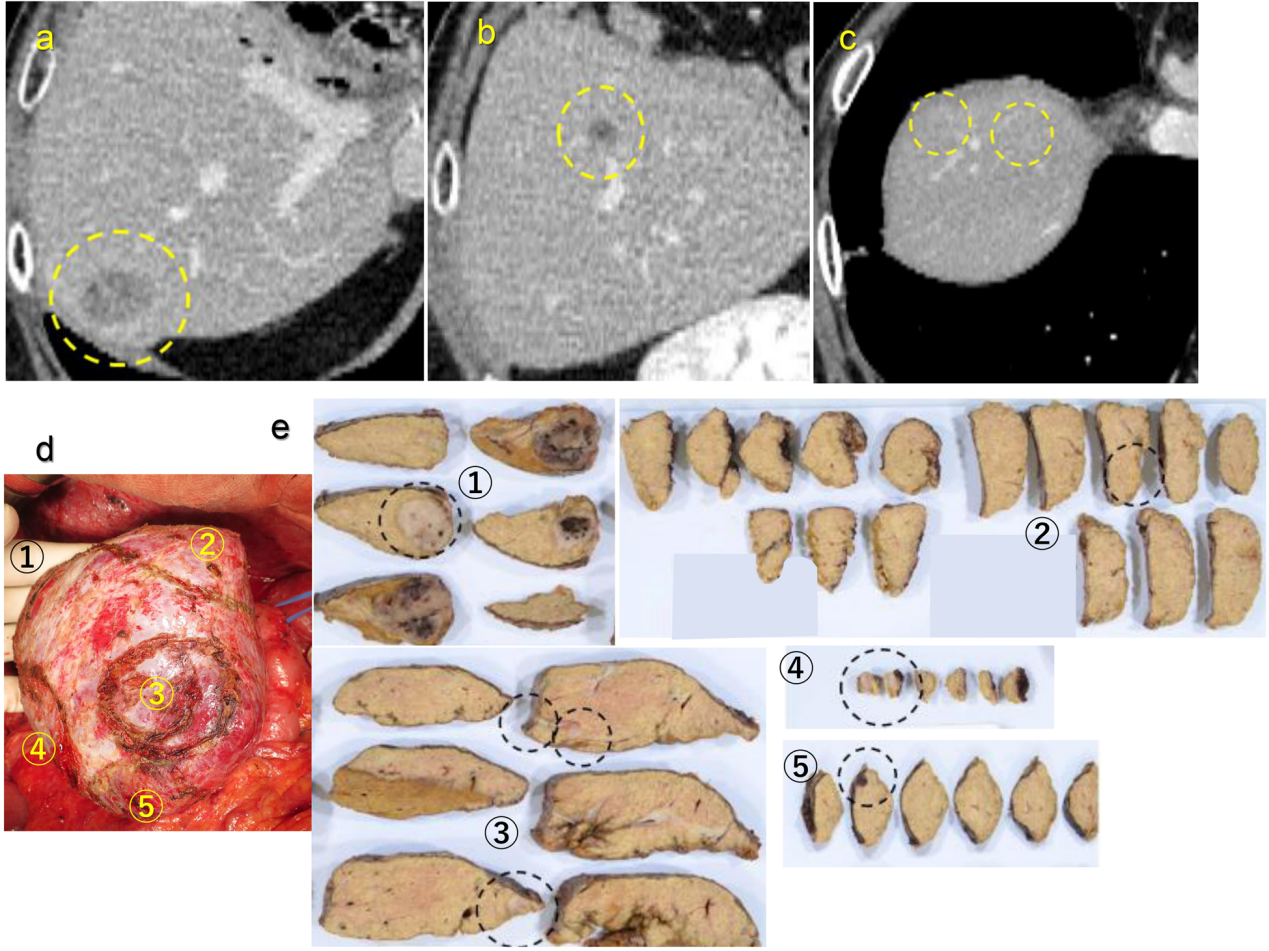
Third operation. **a**–**c** CT detected four liver metastases as recurrence of NET. The tumor was in segments 6, 5, and 8. **d** Intraoperative findings showed three other metastases; five limited liver resections were performed for the tumors. **e** Pathological findings showed seven tumors in the resected specimen which meant that there were 3 other metastases in the resected lesion

Five years and 4 months after the first operation (2 years and 5 months after the second hepatectomy), four liver metastases were detected by CT (Fig. 4a) and magnetic resonance imaging (MRI). In-pentetreotide scintigraphy (OctreoScan) showed uptake in liver tumors. Octreotide long-acting release (LAR) 30 mg was given monthly for 3 months, and the tumor size increased slightly. Then, octreotide LAR was replaced with lanreotide depot/autogel 120 mg at monthly intervals. Seven months later, the tumor grew more but the number of tumors did not change. Then, the third hepatectomy was performed for the four tumors (6 years and 3 months after the first operation). Three other metastases located at segments 5 and 8 were newly detected by intraoperative CEUS, and eventually, five limited liver resections of segments 5, 6, and 8 were performed (Fig. 4d). Pathological findings showed 7 liver metastases with NET G2 recurrence.

**Fig. 5:**
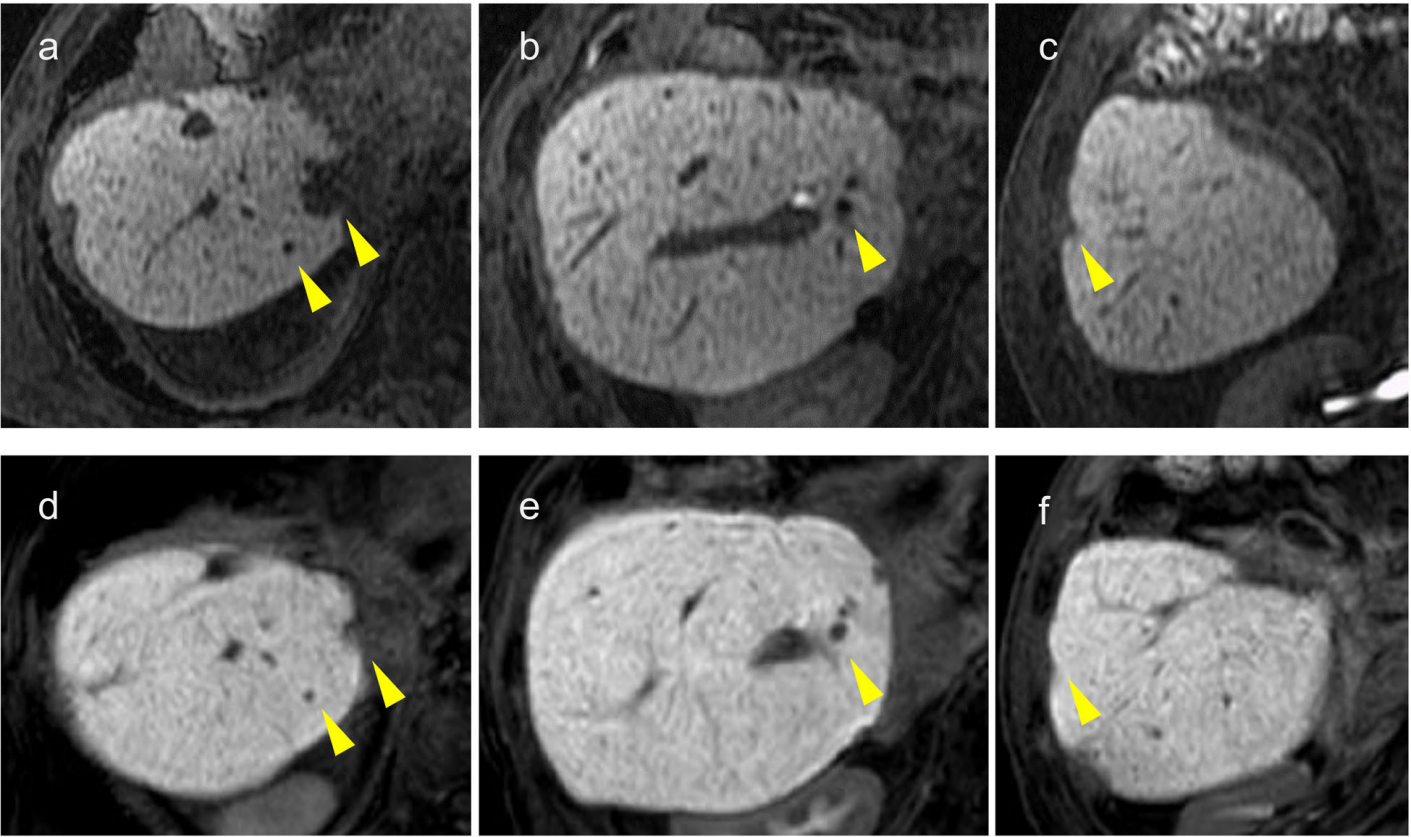
Third recurrence. **a**–**c** Four liver metastases are detected by EOB-MRI. **d**–**f** All tumors shrunk after 6 months of everolimus (10 mg/day) administration. The arrows point the liver metastases detected by EOB-MRI

**Fig. 6:**
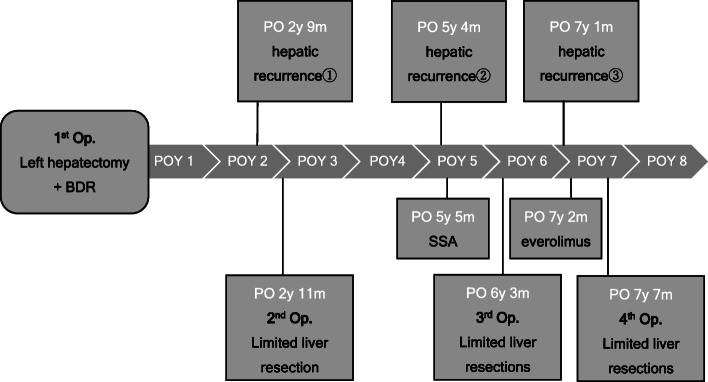
Chronological progress of the case. Operation was performed four times, and long-term survival of 8 years was obtained

Seven years and 1 month after the first hepatectomy (10 months after the third hepatectomy), four liver metastases were detected. After 6 months of everolimus (10 mg/day) administration, all tumors shrank (Fig. 5). Although everolimus seemed to be effective, it was difficult to continue the chemotherapy because of its side effects, such as nausea, anorexia, and taste disorder. In the fourth surgery, no newly detected tumor by CE-IOUS nor pathological findings were noted. After the fourth hepatectomy, the patient had no recurrence and obtained long-term survival for as long as 8 years after the first surgery (Fig. 6).

## Discussion

NET of the extrahepatic bile duct reportedly accounts for only 0.2–2% of primary NET sites [1, 4, 5]. According to a recent analysis of 13,715 carcinoid tumors from the National Cancer Institute database, the incidence of primary extrahepatic bile duct involvement was only 0.32% among all gastrointestinal carcinoid tumors [6]. In a literature review on extrahepatic bile duct NET, 150 cases from 100 articles from 1959 to 2012 were summarized [1]; it showed that the median age was 47 years (range, 6–79 years), with a female (61.5%) predominance. The tumors were symptomatic in 88.5% of the patients. The most common symptoms were jaundice (60.3%), followed by hormone- or vasoactive peptide-related symptoms (9%). The symptoms were mostly related to tumor mass growth, invasion of adjacent structures, or metastases rather than hormone and vasoactive peptide secretion [1]. Surgical excision was considered as the main and only curative treatment for the extrahepatic bile duct NETs. The type of procedure depended on the tumor location. The most frequent procedure was excision of extrahepatic bile duct (62.8%) with portal lymphadenectomy (43.6%). Pancreatoduodenectomy was performed in 19.2% of the patients, hepatectomies or radiofrequency ablation (RFA) in 14.1%, and liver transplantation in 3.85%. In 6.4% of the patients, only biopsies were conducted [1].

Pathologically, NETs may arise from argentaffin or Suschitzky cells, which are believed to be endodermal in origin [7–9]. These cells are present in the gastrointestinal tract, but also exist in extremely low numbers in the bile duct, resulting in the lower occurrence of bile duct NET [8]. Chronic inflammation within the bile duct may cause intestinal metaplasia of the biliary epithelium [10]. The most frequent sites of extrahepatic biliary NETs are the common hepatic duct (19.2%) and the distal common bile duct (19.2%), followed by the middle of the common bile duct (17.9%), the cystic duct (16.7%), and the proximal common bile duct (11.5%) [1].

The liver is the most common site for NET metastasis. At initial diagnosis, about 65–95% of gastroenteropancreatic NET shows hepatic metastasis [11]. Indeed, liver metastases represent the most crucial prognostic factor, irrespective of the primary NET site. In historical series, 5-year survival was 13–54% in patients with hepatic metastases compared with 75–99% in patients without hepatic metastases [12]. In terms of treatment, hepatic resection was associated with high favorable survival compared to chemotherapy [13]. Even cytoreduction hepatectomy was reported to have a comparable outcome to complete resection (R0 or R1) [3]. In a systematic review of the extrahepatic bile duct NETs describing 78 adequately documented cases, metastases were present in one third of all the patients. Lymph node metastasis was 19.5%, followed by liver metastases (16.9%) [1]. Since bile duct NETs or liver metastasis of bile duct NETs is quite rare, standard treatment for this disease has not been established yet.

We reviewed cases involving the bile duct NET G1 or G2 with liver metastasis to study the treatments and prognoses. Literature review using PubMed was employed using the following keywords: [carcinoid] [Neuroendocrine tumor] [Neuroendocrine carcinoma] and [bile duct] [biliary tract] [cystic duct] (Table 1). We identified G1/G2 from the description of mitotic findings or Ki-67. The word “carcinoid” was coined by the pathologist Siegfried Oberndorfer to mean “carcinoma-like” [14]; the description was in reference to the benign behavior of morphologically atypical small bowel tumors [15]. However, the word “carcinoid” led to terminological confusion and diagnostic unreliability because despite the presence of innocuous-appearing cell with uniform nuclei and few mitoses, these tumors sometimes behave malignantly with metastasis, local invasion, and recurrence after resection. Since 2000, the WHO has been revising the gastroenteropancreatic classification to avoid the term “carcinoid” in favor of NET; currently, NET is classified by tumor differentiation, mitotic rate, and Ki-67 in the pathology report [16]. As a result, “carcinoid” nearly represents NET G1/G2, but not the same and has some possibility to contain NET G3 and neuroendocrine carcinoma (NEC) (G3). In this review, the term “carcinoid” was included without NET grading.

**Table 1:** Bile duct NET G1/G2 with liver metastasis

Case	Author/year	Sex	Age	Location	G1/G2	Liver met.	Treatment	Recurrence	Re-treatment	Follow-up time and prognosis
1	D.M. Judge /1976	M	19	PCBD	n/a (carcinoid)	Synchronous	BSC (cholecystectomy, biopsy)			Deceased 4 months after operation
2	Gastinger I /1987	F	65	PCBD	n/a (carcinoid)	Synchronous	Liver resection	n/a		Alive for 5 months
3	B Rodriguez /1991	M	36	CHD-B	n/a (carcinoid)	Synchronous	BSC (exploratory laparotomy)			Deceased 4 days after operation
4	Gembala RB/1993	M	28	RHD-CHD	n/a (carcinoid)	Synchronous	Right trisegmentectomy, BDR, LNR, HJ	n/a		n/a
5	Kopelman D/1996	F	44	CBD	n/a (carcinoid)	Synchronous	Limited liver resection, PPPD	n/a		Alive for 18 months
6	Oikawa I/1998	M	70	CD-CBD	G1	Synchronous	BDR, liver resection (caudal lobe)	Liver	None	Deceased 6 months after operation
7	El Rassi ZS/2004	F	41	Hilar	G1/G2	Synchronous	Left hepatectomy, LNR, HJ	n/a		Alive for 20 years
8	Tzimas GN/2006	F	29	Hilar	n/a (carcinoid)	Synchronous	Left liver, caudate lobe resection, BDR, HJ	1 year and 2 weeks re-op.	Orthotopic liver transplantation	Alive for 3 years (alive for 2 years after 2nd operation)
9	Honda H/2006	M	76	CBD	G1/G2	Metachronous	PD	Liver (8 months after op.)	n/a	Alive for more than 8 months after operation
10	Ferrone CR/2007	M	52	RHD-H	G1	Synchronous	Right trisegmentectomy, BDR, LNR	n/a		n/a
11	Felekouras E/2009	M	60	CBD	G2	Metachronous	BDR, HJ	Liver (1 year after op.)	RFA	Alive for more than 1 year after second treatment

*CBD* common bile duct, *CHD* common hepatic duct, *RHD* right hepatic duct, *CD* cystic duct, *PCBD* proximal common bile duct, *CHD-B* common hepatic duct bifurcation, *RHD-H* right hepatic duct–hilar, *PD* pancreatoduodenectomy, *PPPD* pylorus-preserving pancreateduodenectomy, *BDR* bile duct resection, *LNR* lymph node resection, *HJ* hepaticojejunostomy

Eighty-four cases from 76 articles were sorted under bile duct NETG1/G2 and carcinoid. Among them, 11 cases are with liver metastases. Table 1 summarizes these bile duct NET G1/G2 and carcinoid with liver metastasis [10, 17–25]. The female to male ratio was 4/7 with a median age of 44 years (ranging from 19 to 76 years). The most frequent sites were the common bile duct (36%) followed by hilar (18%) and proximal common bile duct (18%). Nine patients had synchronous liver metastasis, while two had metachronous ones. In the nine patients with synchronous liver metastasis, seven underwent hepatectomy and two were judged to have unresectable metastases. After hepatectomy for synchronous liver metastasis, one of these seven patients developed recurrence of liver metastasis and died 6 months after the operation. In the two cases with metachronous liver metastasis, one was diagnosed with liver metastasis 8 months after the first operation without detailed reports of treatment for the recurrence. The other patient had liver metastasis 1 year after the primary resection and underwent CT-guided percutaneous RFA. Although the prognosis had not been well documented in most cases, median follow-up time was 7 months (ranging from 0 to 240 months). One patient with curative surgery obtained long-term survival of 20 years [21].

In our case, we performed upfront hepatectomy for the first liver metastases, since CT and MRI showed solitary tumor (although the number of tumors increased during surgery). In the second recurrence, multiple liver metastases were detected by CT and MRI, which were avid on the OctreoScan. To confirm that the number of tumors was not increasing rapidly, or the metastases were limited to the liver, we chose somatostatin analogues (SSA) for the second liver recurrence, including octreotide LAR and lanreotide depot/autogel. According to the previous report, SSA achieved stable disease in 87% of the patients and a partial response of 5–8% for gastroenteropancreatic NET [26]. This effect is limited from 6 to 18 months [27]. In our case, octreotide LAR was given for 3 months followed by lanreotide depot/autogel for 7 months. Although the size of tumors increased, the number of tumors did not change, and third hepatectomy was indicated. For the third recurrence (before fourth hepatectomy), we chose everolimus instead of SSA. Six months of everolimus caused the side reactions, so the fourth operation was planned. The main reason for chemotherapy induction for the multiple liver metastases was to see whether the tumors got worse rapidly or slowly. In the second recurrence, the chemotherapy could not give good response (the size of tumor increased); the number of tumors did not increase for more than 10 months. Then, the surgery was planned. In the third recurrence, although the tumors shrank, chemotherapy could not be continued because of the side effects, and surgery was planned. In both situations, chemotherapy took an important role of watching disease control.

It is important to resect as many tumors as possible for long-term survival. CE-IOUS may be effective to find new tumors that are difficult to identify via CT, EOB-MRI, or CEUS before surgery [12]. CEUS was routinely performed a day before the surgery. In the repeat hepatectomy cases, although we could not find new tumors by the CEUS before the surgery, we identified new tumors by CE-IOUS during the second and third surgeries. Hereby, CE-IOUS was quite useful to detect new liver metastases from NET.

## Conclusion

Liver resection can contribute to long-term survival in the context of a multidisciplinary approach in patients with liver metastases of NET. In selected patients, repeat hepatectomy should be considered for liver metastases originating from bile duct NET G2 as well as other gastroenteropancreatic NETs.

## Data Availability

The datasets used and/or analyzed during the current study are available from the corresponding author on reasonable request.

## Abbreviations

CT: Computed tomography
CEUS: Contrast-enhanced ultrasonography
CE-IOUS: Contrast-enhanced intraoperative ultrasound
ERCP: Endoscopic retrograde cholangiopancreatography
EOB-MRI: Gadolinium ethoxybenzyl diethylenetriamine penta-acetic acid-enhanced magnetic resonance imaging
NET: Neuroendocrine tumor
RFA: Radiofrequency ablation
SSA: Somatostatin analogues

## References

[1] Michalopoulos N, Papavramidis TS, Karayannopoulou G, Pliakos I, Papavramidis ST, Kanellos I (2014). Neuroendocrine tumors of extrahepatic biliary tract. Pathol Oncol Res..

[2] Godwin JD (1975). Carcinoid tumors. An analysis of 2,837 cases. Cancer..

[3] Frilling A, Modlin IM, Kidd M, Russell C, Breitenstein S, Salem R (2014). Recommendations for management of patients with neuroendocrine liver metastases. The Lancet Oncology..

[4] Chamberlain RS, Blumgart LH (1999). Carcinoid tumors of the extrahepatic bile duct. A rare cause of malignant biliary obstruction. Cancer..

[5] Albores-Saavedra J, Batich K, Hossain S, Henson DE, Schwartz AM (2009). Carcinoid tumors and small-cell carcinomas of the gallbladder and extrahepatic bile ducts: a comparative study based on 221 cases from the Surveillance, Epidemiology, and End Results Program. Ann Diagn Pathol..

[6] Modlin IM, Lye KD, Kidd M (2003). A 5-decade analysis of 13,715 carcinoid tumors. Cancer..

[7] Abe T, Nirei A, Suzuki N, Todate Y, Azami A, Waragai M (2017). Neuroendocrine tumor of the extrahepatic bile duct: a case report. Int J Surg Case Rep..

[8] Dancygier H, Klein U, Leuschner U, Hubner K, Classen M (1984). Somatostatin-containing cells in the extrahepatic biliary tract of humans. Gastroenterology..

[9] Judge DM, Dickman PS, Trapukdi S (1976). Nonfunctioning argyrophilic tumor (APUDoma) of the hepatic duct: simplified methods of detecting biogenic amines in tissue. Am J Clin Pathol..

[10] Barrón-Rodríguez LP, Manivel JC, Méndez-Sánchez N, Jessurun J (1991). Carcinoid tumor of the common bile duct: evidence for its origin in metaplastic endocrine cells. Am J Gastroenterol..

[11] Saxena A, Chua TC, Sarkar A, Chu F, Liauw W, Zhao J (2011). Progression and survival results after radical hepatic metastasectomy of indolent advanced neuroendocrine neoplasms (NENs) supports an aggressive surgical approach. Surgery..

[12] Kulke MH, Benson AB, Bergsland E, Berlin JD, Blaszkowsky LS, Choti MA (2012). Neuroendocrine tumors. J Natl Compr Canc Netw..

[13] Fairweather M, Swanson R, Wang J, Brais LK, Dutton T, Kulke MH (2017). Management of neuroendocrine tumor liver metastases: long-term outcomes and prognostic factors from a large prospective database. Ann Surg Oncol..

[14] Modlin IM, Shapiro MD, Kidd M (2004). Siegfried Oberndorfer: origins and perspectives of carcinoid tumors. Hum Pathol.

[15] Rosai J (2011). The origin of neuroendocrine tumors and the neural crest saga. Mod Pathol..

[16] Kloppel G (2011). Classification and pathology of gastroenteropancreatic neuroendocrine neoplasms. Endocr Relat Cancer..

[17] Gastinger I, Schutze U, Beetz G, Lippert H (1987). Obstructive jaundice caused by a carcinoid tumor of the hepatocholedochal duct. Zentralbl Chir..

[18] Gembala RB, Arsuaga JE, Friedman AC, Radecki PD, Ball DS, Hartman GG (1993). Carcinoid of the intrahepatic ducts. Abdom Imaging..

[19] Kopelman D, Schein M, Kerner H, Bahuss H, Hashmonai M (1996). Carcinoid tumor of the common bile duct. HPB Surg..

[20] Oikawa I, Hirata K, Katsuramaki T, Mukaiya M, Sasaki K, Satoh M (1998). Neuroendocrine carcinoma of the extrahepatic biliary tract with positive immunostaining for gastrin-releasing peptide: report of a case. Surg Today..

[21] El Rassi ZS, Mohsine RM, Berger F, Thierry P, Partensky CC (2004). Endocrine tumors of the extrahepatic bile ducts. Pathological and clinical aspects, surgical management and outcome. Hepatogastroenterology..

[22] Tzimas GN, Vali K, Deschênes M, Marcus VA, Barkun JS, Tchervenkov JI (2006). Liver transplantation for metastases from a bile duct carcinoid. HPB (Oxford)..

[23] Honda H, Hayashi S, Sekiguchi Y, Tsukadaira T, Nakamura K (2006). A case of the extrahepatic bile duct carcinoid tumor. Nihon Shokakibyo Gakkai Zasshi..

[24] Ferrone CR, Tang LH, D'Angelica M, DeMatteo RP, Blumgart LH, Klimstra DS (2007). Extrahepatic bile duct carcinoid tumors: malignant biliary obstruction with a good prognosis. J Am Coll Surg..

[25] Felekouras E, Petrou A, Bramis K, Prassas E, Papaconstantinou I, Dimitriou N (2009). Malignant carcinoid tumor of the cystic duct: a rare cause of bile duct obstruction. Hepatobiliary Pancreat Dis Int..

[26] Massironi S, Conte D, Rossi RE (2016). Somatostatin analogues in functioning gastroenteropancreatic neuroendocrine tumours: literature review, clinical recommendations and schedules. Scand J Gastroenterol..

[27] Oberg KE (2010). Gastrointestinal neuroendocrine tumors. Ann Oncol.

